# ﻿Three new species of *Pleurothallis* (Orchidaceae) from Costa Rica and Panama, with a note on asexual reproduction by prolification in Pleurothallidinae

**DOI:** 10.3897/phytokeys.256.140316

**Published:** 2025-05-29

**Authors:** Adam P. Karremans, Franco Pupulin, John Gange, Diego Bogarín

**Affiliations:** 1 Centro de Investigación Jardín Botánico Lankester, Universidad de Costa Rica, P.O. Box 302-7050, Cartago, Costa Rica Universidad de Costa Rica Cartago Costa Rica; 2 Harvard University Herbaria, Cambridge, Massachusetts 02138, USA Harvard University Herbaria Massachusetts United States of America; 3 The Marie Selby Botanical Gardens, Sarasota, Florida 34236, USA The Marie Selby Botanical Gardens Florida United States of America; 4 Pringle Herbarium, Department of Plant Biology, University of Vermont, Burlington, Vermont 05405, USA University of Vermont Vermont United States of America; 5 Naturalis Biodiversity Center, Leiden, 2333 CR, Netherlands Naturalis Biodiversity Center Leiden Netherlands; 6 Herbario UCH, Universidad Autónoma de Chiriquí, 0427, David, Chiriquí, Panama Universidad Autónoma de Chiriquí Chiriquí Panama

**Keywords:** Costa Rica, epiphytic orchids, Panama, prolific ramicaul, Talamanca range, vegetative growth

## Abstract

Three new species of *Pleurothallis*, each exhibiting prolific vegetative growth, are described from Costa Rica and western Panama. Prolification refers to the development of a vegetative bud from the axil of a bract within the floral meristem, a frequent condition in the Pleurothallidinae, where it manifests as a new ramicaul developed from the apex of a previous ramicaul. Prolification can be either constitutive or facultative, the latter occurring mostly under stress-induced, non-optimal growing conditions. The three new species are found at high elevations, between 1400 and 2550 m, in humid, dense, mossy conditions on the Talamanca range, where they naturally produce prolific growths on the ramicaul apex that sever from the plant with time. *Pleurothallismatrisilvae***sp. nov.** superficially resembles *P.bothros*, but may be distinguished by the prolific habit, thin ramicauls, typically bearing 1–2 open flowers, the longer flower segments, the lanceolate petals, and the black flecks on the pedicel, ovary, and external surface of the sepals. *Pleurothallispridgeoniana***sp. nov.** is similar to *P.vinealis* but distinguished by the significantly shorter plants and ramicauls, the much smaller yellow flowers with a rose to purple suffusion, and the triangular-ovate lip with glandular margins and lacking a central sulcus. *Pleurothalliswinkeliana***sp. nov.**, closely resembles *P.longipetala*, but differs by the occasionally prolific plant that produces clumps of ramicauls, the comparatively broader, cordate leaves with overlapping basal lobes, the smaller flower, and the pendent lip, perpendicular to the column.

## ﻿Introduction

*Pleurothallis* R.Br. (Aiton 1813: 211), currently includes around 550 accepted species, being surpassed only by *Lepanthes* Sw. (Swartz 1799a: 85), *Masdevallia* Ruiz & Pavón (Ruiz and Pavón 1794: 122), and *Stelis* Sw. (Swartz 1799b: 239) as the most species-rich genus in the Pleurothallidinae (Karremans 2016; Karremans and Vieira-Uribe 2020). They are distributed from Mexico through Central America and the Antilles to the Andean and Guayanan countries of South America, where they reach their highest diversity (Luer 2005). In Costa Rica, *Pleurothallis* is the fourth most species-rich orchid genus with 63 species and four natural hybrids recognized by Pupulin et al. (2023). Although some highly morphologically distinctive *Pleurothallis* species were recently described from Costa Rica (e.g. Karremans and Bogarín 2011), most novelties are recognized when species complexes are carefully monographed, as it happened in the *Pleurothallisbothros*Luer (1996: 70), *P.cardiothallis* Rchb.f. (Reichenbach 1857: 158) and *P.phyllocardia* Rchb.f. (Reichenbach 1866: 97) groups (Pupulin et al. 2017a, 2017b, 2021; Karremans and Jiménez 2018). Despite thorough studies, Pupulin et al. (2023) noted that the number of *Pleurothallis* species in Costa Rica with a small habit and flowers, colloquially known as ‘frogs’, will likely quadruple in the near future. One such species was described as *P.trigyna* Pupulin (Belfort-Oconitrillo et al. 2024: 173), and here we describe three additional *Pleurothallis* species from Costa Rica and western Panama.

**Figure 1.**
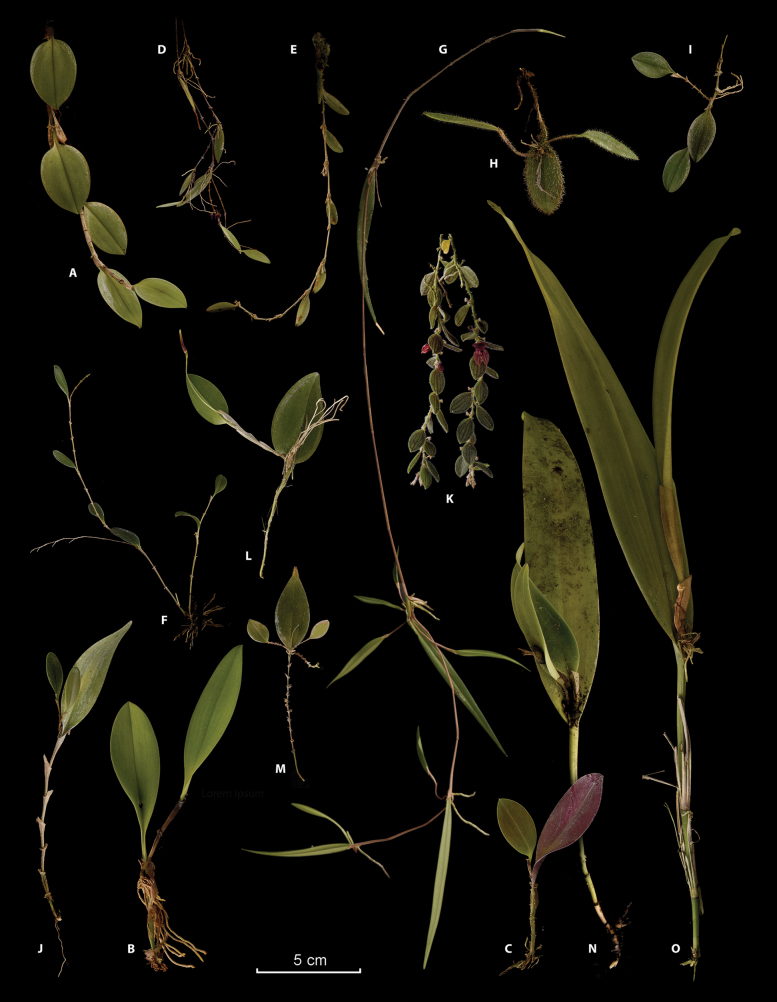
Prolific ramicauls in species across Pleurothallidinae**A***Zootrophionmachaqwayi* (*JBL-4585*) **B***Z.vulturiceps* (*Pupulin 3960*) **C***Z.gracilentus* (*Pupulin 5220*) **D***Lepanthopsisprolifera* (*JBL-458091*) **E***Karmachaetoglossa* (*JBL-45847*) **F***Karma* sp. (*Álvarez 1444*) **G***Myoxanthusscandens* (*Rojas-Alvarado 1621*) **H***Dresslerellapilosissima* (Pupulin 8070) **I***Trichosalpinx* sp. (*Bogarín 11972*) **J***Trichosalpinxblaisdellii* (Fernández 123) **K***Andinia* sp. (*CIOA-001590*) **L***Restrepiatrichoglossa* (*Pupulin 7889*) **M***Lepanthes* sp. (*Álvarez 1466*) **N***Echinosepalauncinata* (*Chinchilla 1026*) **O***E.longipedunculata* (*Rojas-Alvarado 570*). Photographs and digital composition by FP.

**Figure 2.**
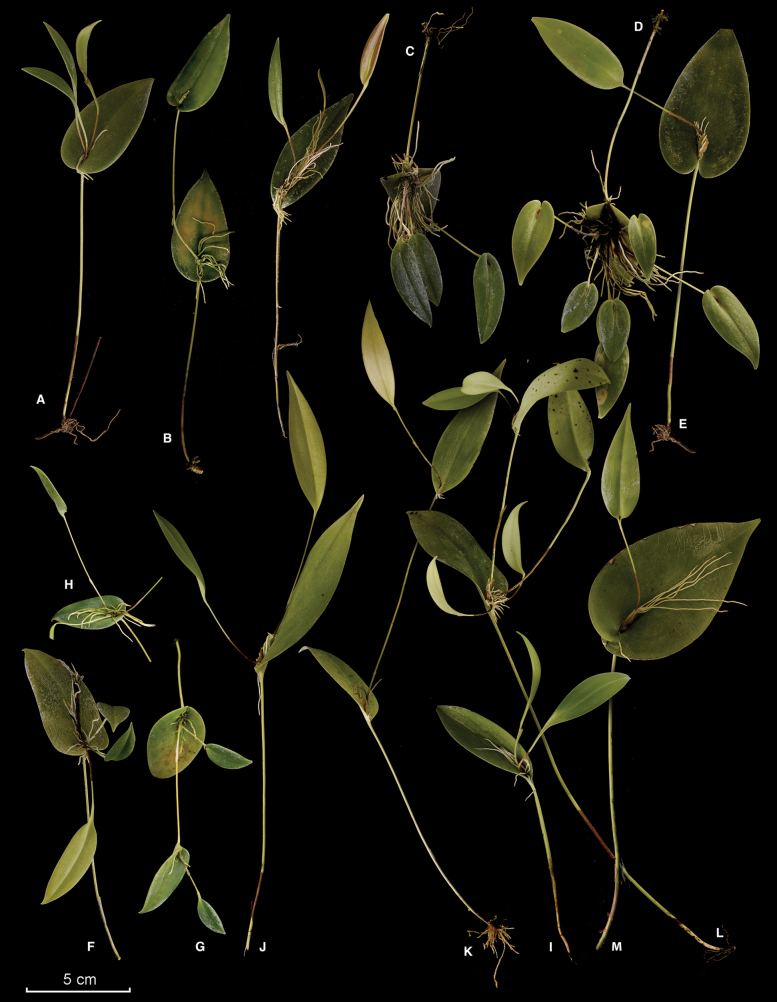
Prolific ramicauls in species of *Pleurothallis*. *Macrophyllae-fasciculate* group **A***P.matrisilvae* (*Pupulin 8951*) **B***P.phyllocardioides* (*Chinchilla 4380*) **C***P.homalantha* (*Pupulin 9040*) **D***Pleurothallis* sp. (*Fernández 250*) **E***P.winkeliana* (*Chinchilla 3408*) **F***P.palliolata* (*Bogarín 9101*) **G, H***P.pridgeoniana* (*Chinchilla 2865*). *Macrophyllae-racemosae* group. *Ancipitae* group **I***P.eumecocaulon* (*Rojas-Alvarado 1556*) **I***P.crocodiliceps* (*Pupulin 5783). Pleurothallis* group **J***P.ruscifolia* (*Pupulin 7013*) **K**Pleurothalliscf.ruscifolia (*Smith 261*) **L***Pleurothallis* sp. (*Bogarín 5304*) **M***P.rowleei* (*Pupulin 4352*). Photographs and digital composition by FP and APK (**G, H**).

Vegetative morphology in *Pleurothallis* is somewhat uniform, members of this genus usually form caespitose plants with ramicauls emerging from the rhizome. But the three species treated here stand out for exhibiting a prolific growth habit. Prolification refers to the phenomenon in certain plants where the floral meristem develops a vegetative bud from the axil of a bract (Bell and Bryan 2008). In orchids, these clones, named keikis in horticulture, are usually associated with adventitious roots and can grow independently if shed or removed from the inflorescence axis, resulting in an asexual reproduction method (Rojas-Alvarado et al. 2021; Rojas-Alvarado and Karremans 2024). Prolification is frequent across Pleurothallidinae, where it often involves the formation of a ramicaul, on the apex of a previous ramicaul, occasionally forming chains (Figs 1, 2). In some Pleurothallidinae, prolification is a constitutive feature, representing the standard condition for the species or group of species. This is true for a few species in *Pleurothallis* and *Stelis*, to all species of MyoxanthusPoepp. & Endl.sect.Scandentia Luer (Luer 1992: 5), and *Karma* Karremans (Karremans 2023: 62), and to most species of *Andinia* (Luer) Luer (Luer 2000: 5), among others, which form pendent chains of superposed stems. The vast majority of pleurothallids, however, exhibit facultative prolification, occurring only occasionally, mostly under stress-induced, non-optimal, growing conditions (Rojas-Alvarado et al. 2021; Rojas-Alvarado and Karremans 2024). Most constitutively prolific Pleurothallidinae form long chains of ramicauls that lack roots and do not naturally detach from the plant (Figs 1A, E–G, K). In contrast, the prolific growth of certain *Pleurothallis* species produces multiple roots and ramicauls that can naturally sever from the plant with age. Strictly prolific species of *Pleurothallis* include *P.killipii* Garay (Garay 1956: 254), *P.lopezii* Luer & R.Escobar (Luer and Escobar 1998: 102), *P.palliolata* Ames (Ames 1922: 86) (Fig. 2F) and *P.vinealis* Luer & R.Escobar (Luer and Escobar 1986: 30). These are all high elevation species, often growing at around 2000 m, and up to 3000 m in elevation, where they are found in humid, dense, mossy vegetations. In the field, large plants with such a habit are typically found surrounded by independently growing plantlets, which arise by prolification as a means of asexual reproduction (Rojas-Alvarado and Karremans 2024).

While preparing a monograph of the genus *Pleurothallis* for Costa Rica, we found three unidentified taxa with a prolific habit growing at high-elevations in the Talamanca mountain range. These novelties belong to Pleurothallissubsect.Macrophyllae-*Fasciculatae* [= Acroniasect.Macrophyllae-*Fasciculatae* (Lindl.) Luer] as defined by Luer (Luer 1988; 2005), but may not be closely related. Unfortunately, no broad phylogeny of *Pleurothallis* is currently available. Studies including only a small sampling fraction of the genus show that *Pleurothallis* is monophyletic as currently defined (Karremans 2016), but sections and subsections are not monophyletic, including the paraphyletic Pleurothallissubsect.Macrophyllae-*Fasciculatae* (Karremans et al. 2013; Pérez-Escobar et al. 2017; Chumová et al. 2021). How these species groups interrelate remains to be shown. Although we can only speculate about their phylogenetic relationships, we are confident the taxa described here represent previously unknown entities. Therefore, we formally describe and illustrate them, based on field-collected and cultivated specimens.

## ﻿Methods

Plant collection and documentation followed the methods described by Pupulin et al. (2021). Living plants intended for this study were collected between 2010 and 2024 and cultivated at Lankester Botanical Garden (JBL, its acronym in Spanish), University of Costa Rica. At JBL plants were individually labelled and cultivated, and field data was recorded and stored in the general databases. Living plants from Costa Rica and Panama were photographed using Nikon 7100, 810, and 850 cameras, and the herbarium specimens were prepared from living plants and deposited in the liquid or dried collections at JBL and USJ (acronyms following Thiers 2025, continuously updated). Images were optimized through post-processing with Adobe Photoshop 2024, and the Lankester Composite Digital Plates (LCDP) and other comparative plates of flowers were prepared using the same software. The cited georeferenced occurrences were plotted onto a shaded relief base map using the free and open-source QGIS 3.34.

Standard characters states in *Pleurothallis*, such as green leaves, glabrous perianth parts, or regular and straight margins, are not mentioned expressly in the description. Terminology for the description of the inflorescences follows Rojas-Alvarado and Karremans (2024). Descriptions are based on the specimens indicated at the end of each description account. For consistency, we adopt the term ramicaul proposed by Stern and Pridgeon (1984), to refer to the leaf-bearing stem in the Pleurothallidinae. Although we acknowledge the concerns raised by Rasmussen (1985), who argued that this structure is not a true branch in the botanical sense (from Latin *ramus* = branch, *caulis* = stem), but rather a continuation of the main axis in sympodial orchids, the term ramicaul has been widely adopted in most of the taxonomic literature on Pleurothallidinae, particularly by Luer (1988, 1992, 1996, 2000, 2005) and subsequent contemporary works (Pupulin et al. 2017a, 2017b, 2021; Karremans and Jiménez 2018; Sierra-Ariza et al. 2022; Moreno et al. 2023). Additionally, we find the term useful for distinguishing different elements within the prolific growth system of Pleurothallidinae species. This should not be confused with the terms primary ramicaul and secondary ramicaul, which are used here to differentiate the elements that compose a prolific growth system. As used here, a primary ramicaul refers to the shoot that originates directly from the primary stem (the rhizome), while a secondary ramicaul refers to a stem that develops from the apical meristem of a ramicaul.

## ﻿Taxonomic treatment

### ﻿ Pleurothallis matrisilvae

Taxon classificationPlantaeAsparagalesOrchidaceae

Karremans, J.Gange & Pupulin sp. nov.

C0944F9F-B518-5453-9722-7835ABC5B2ED

urn:lsid:ipni.org:names:77362475-1

#### Type.

**Figure 3.**
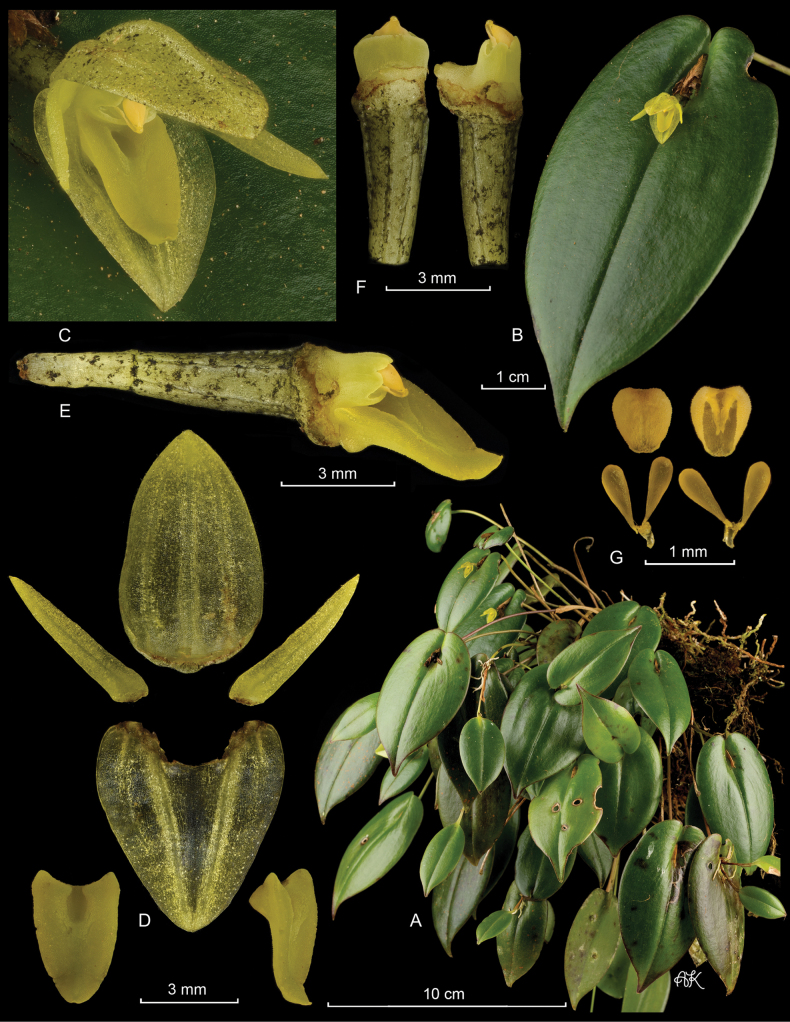
*Pleurothallismatrisilvae* Karremans, J.Gange & Pupulin **A** habit, showing some ramicauls bearing flowers and others bearing new growths **B** single leaf with flower **C** flower **D** dissected perianth (dorsal sepal, two lateral petals, lateral sepals fused into a synsepal and two views of the lip) **E** column and lip, lateral view **F** column and ovary, lateral and ventral views **G** two views of the anther cap and pollinarium. LCDP prepared by APK based on *Karremans 9036*.

Costa Rica. • Cartago: El Guarco, San Isidro, Madreselva, Tres de Junio, Carretera Interamericana Sur, 9°40'31"N, 83°53'33"W, 2530 m, bosque pluvial montano, en bosque secundario de robles a orillas de la carretera, 5 March 2022, fl. in cult. 8 March 2022, *A.P. Karremans & J. Gange 9036* (holotype: JBL-spirit, M0058!; Fig. 3).

#### Diagnosis.

The flower is superficially similar to that of *Pleurothallisbothros*, however the new species may be distinguished by the prolific habit (vs. non-prolific), ramicauls linear, very thin throughout (vs. ramicauls subclavate, notably thickened apically), bearing 1–4 open flowers (vs. many, usually +10, simultaneous flowers), the longer flower segments (dorsal sepal 7.4–7.6 vs. 6.5–6.6 mm, lateral sepals 6.6 vs. 6.0 mm, petals 5.6–5.7 vs. 3.8–4.0 mm, lip 4.1–4.3 vs. 3.1–3.5 mm), the lanceolate petals (vs. oblong) and the black flecks on the pedicel, ovary, and external surface of the sepals (vs. no flecks).

#### Description.

Epiphytic, caespitose, constitutively prolific, suberect to erect herbs, up to 30 cm tall. ***Roots*** flexuous, thin, ca. 1 mm in diameter, densely spaced, appearing fasciculate. ***Ramicauls*** erect to suberect, slender, up to 28 cm long, covered by tubular sheaths close to the base, tightly adpressed, up to 5 cm long. ***Leaves*** spreading, glossy, dark green, coriaceous, sessile, ovate-cordate with downturned margins, acuminate, blades of the primary ramicaul 4.0–7.5 × 2.0–3.8 cm. ***Inflorescences*** persistent, forming few to several successive multi-flowered coflorescences, each producing a single open flower, up to 4 different coflorescences bearing simultaneous single flowers, typically 1 or 2, subtended by a nearly prostrate or suberect spathe which appears deeply torn during and after anthesis, ca. 1 cm long; ***pedicels*** terete, pale gray-yellow, with irregular black flecks. ***Ovary*** clavate, 4.5–4.7 mm long, gray-green and suffused with yellow, similarly marked with black. Flowers spreading, yellow. ***Dorsal sepal*** elliptic-ovate, slightly concave, obtuse, 3-veined, with irregular black flecks on its adaxial surface, 7.4–7.6 × 4.5 mm, apiculate. ***Lateral sepals*** connate in an oblong, slightly concave synsepal, obtuse, 4-veined, 6.6 × 5.8–5.9 mm. ***Petals*** linear-lanceolate, oblique, acute, 1-veined, 5.6–5.7 × 1.0–1.1 mm. ***Lip*** triangular-ovate, resting on the synsepal, 4.1–4.3 × 2.7–2.8, margins raised, apex slightly recurved, acute, glenion a deep cavity between the raised lateral margins. ***Column*** straight, transversely subrectangular, 1.7–1.9 mm long, apically 2.1 mm wide, with a ca. 1 mm column foot, the anther and stigma apical. ***Anther cap*** ovate, cucullate, obtuse at the base, bilobed apically, 2-celled. ***Pollinarium*** composed of 2, narrowly ovate-pyriform pollinia, connected to a liquid, drop-like viscidium. ***Fruits*** and ***seeds*** unknown. This description is based on *A.P. Karremans & J. Gange 9036, D.E. Mora s.n.*, and *D. Bogarín et al. 13652*.

#### Additional specimens examined.

**Figure 4.**
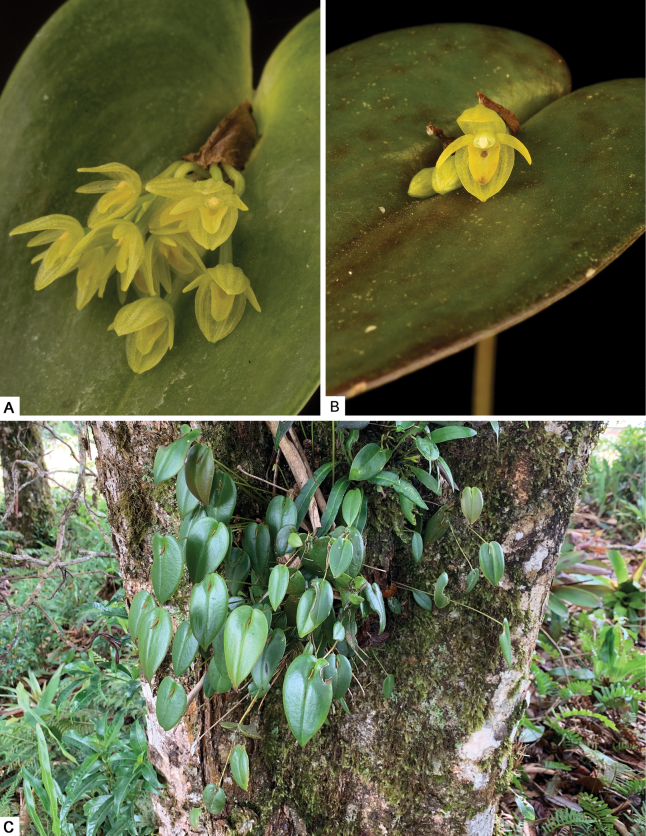
*Pleurothallismatrisilvae* Karremans, J.Gange & Pupulin composite **A** the florally similar *P.bothros* (*JBL-sn*) **B** bearing a single open flower and several developing buds (*Bogarín 13652*) **C** epiphytic plant in its natural habitat in Madreselva on the central Talamanca range, showing the prolific growth and asexual production of new units. Photographs by APK.

Costa Rica. • Cartago: Cordillera de Talamanca, alt. 2400 m, *D.E. Mora s.n.* (USJ!). Cartago: El Guarco, Cañón, Bajo Gloria, Centro Ecoturístico Los Robles, inicio del sendero Danta, 9°42'14.97"N, 83°54'55.96"W, 2332 m, epífitas en árboles alrededor de los senderos, bosque muy húmedo montano bajo, 12 February 2022, fl. in cult. 19 April 2022, *D. Bogarín 13652, S. Bogarín, M. Bonilla & O.A. Pérez-Escobar* (JBL-Spirit, E1544!; Fig. 4B). • San José-Cartago: Dota-El Guarco, Copey-Cañon, ca. 100 m deviation point in front of the Liceo Rural of Cañon, slopes of Cerro Artieda, 9°40'52.44"N, 83°55'10.04” W, 2545 m, epiphytic on scattered trees in pastures and remnants of secondary woods, 3 March 2022, fl. in cult. 21 April 2022, *F. Pupulin 8951 & D. Bogarín* (JBL-spirit, E1545!).

#### Eponymy.

From the Latin “*matrisilva*”, mother-forest, honoring the locality Madreselva, where the type specimen was collected.

#### Phenology.

Flowering has been recorded at least from November to April, which approximately corresponds to the end of the rainy season and the first months of the dry season in Costa Rica.

#### Distribution and ecology.

Currently known only from central Costa Rica, where the species is found growing either epiphytically, mainly on oaks, or terrestrially, on the leaf mulch rich ground, in montane or cloud forests at high elevations around 2300–2550 m (Fig. 4C). *Pleurothallismatrisilvae* is not a particularly rare species, but all currently known specimens were collected in the same general area on the Cordillera de Talamanca in the province of Cartago.

#### Notes.

Contrary to the other species described here, *Pleurothallismatrisilvae* is typically prolific when growing as an epiphyte, while it is mostly non-prolific when found growing on top of organic matter on the ground. The new species is morphologically similar to *P.bothros* and its relatives. It, however, grows at higher elevations and is distinguished by the often-prolific habit, the thin ramicauls and especially by having mainly one or two flowers open at once, which is unlike all other species in the *P.bothros* group (Karremans and Jiménez 2018). It is most similar to *Pleurothallisbothros*, which is endemic to the Cordillera de Guanacaste and Tilarán in the northern provinces of Alajuela, Guanacaste, and Puntarenas in Costa Rica, where it grows at mid elevations between 750 and 1750 m. The other two members of the *P.bothros* group, *P.hawkingii* Karremans & J.E.Jiménez and *P.vide-vallis* Karremans & J.E.Jiménez can be easily separated from *P.matrisilvae* by the non-prolific habit (vs. prolific), three or more flowers opening simultaneously (vs. typically one or two), their pale yellow flowers suffused with a pink (vs. solid yellow), the broad, oblique petals (vs. narrow, straight), and the proportionally much larger lip. Both are only known from the Cordillera de Guanacaste in northern Costa Rica (Karremans and Jiménez 2018).

### ﻿ Pleurothallis pridgeoniana

Taxon classificationPlantaeAsparagalesOrchidaceae

Karremans, Bogarín & Pupulin sp. nov.

A8E88CED-A6B4-5C96-82E1-EF16E883DE23

urn:lsid:ipni.org:names:77362476-1

#### Type.

**Figure 5.**
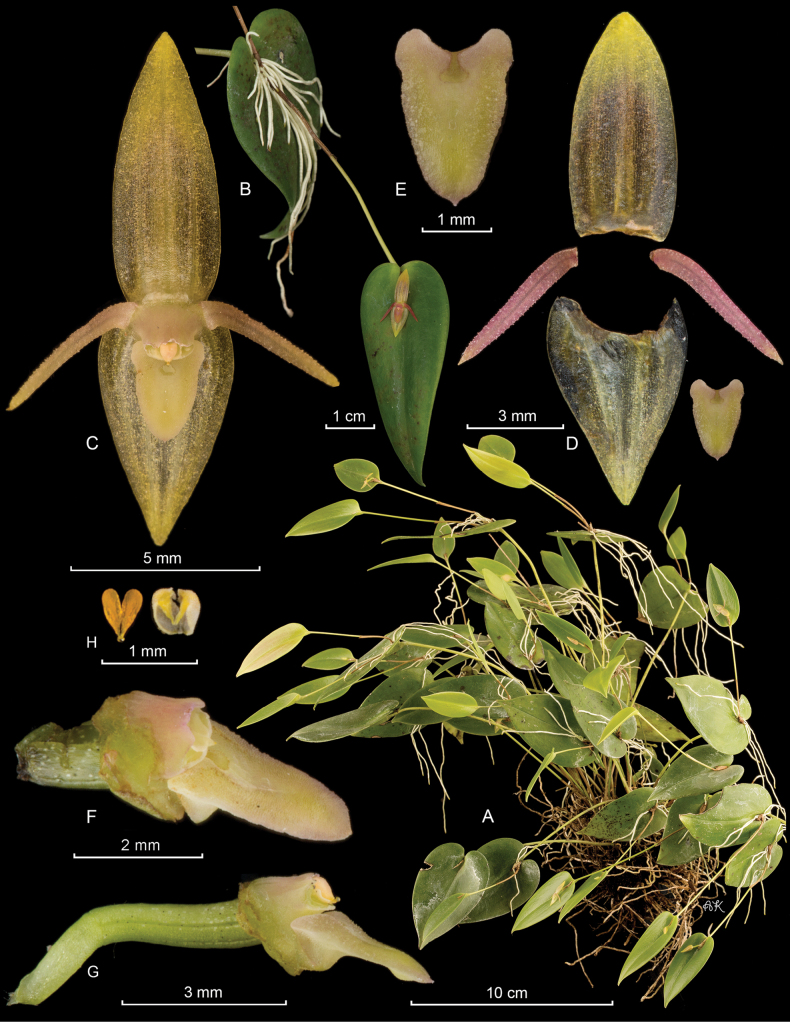
*Pleurothallispridgeoniana* Karremans, Bogarín & Pupulin **A** habit showing the typical prolific vegetative growth **B** prolific growth featuring a single flower on a secondary ramicaul **C** flower **D** dissected perianth (dorsal sepal, two lateral petals, two lateral sepals fused into a synsepal and a view of the lip) **E** lip **F** column and lip, lateral view **G** column, lip and ovary, lateral view **H** anther cap and pollinarium. LCDP prepared by APK based on *Karremans 7600* (**A, C–H**) and *Bogarin 8632* (**B**).

Costa Rica. Puntarenas: Buenos Aires, Buenos Aires, camino a Cerros Utyum, 9°18'04.93"N, 83°12'51.59"W, 2157 m, bosque pluvial montano bajo, epífitas en bosque secundario, 16 January 2017, fl. in cult. 20 December 2017, *A.P. Karremans, D. Bogarín, M. Cedeño, I. Chinchilla, M. Díaz, E. Kaes, P. Lehmann & O. Zúñiga 7600* (holotype: JBL-spirit, E1514!; isotype, USJ!; Fig. 5).

#### Diagnosis.

Vegetatively similar to *P.vinealis*, but distinguished by the significantly shorter plants, up to 30 cm long (vs. exceeding 1 m), the shorter ramicauls 7.5–26.0 cm (vs. up to 40 cm long), the significantly smaller dorsal sepal (6.8–7.0 × 3.3–3.4 mm vs. 11–14 × 4.5–5.5 mm), synsepal (5.5–6.5 × 4.1–4.2 mm vs. 11–14 × 4.5–5.5 mm), and lip (2.4–2.6 × 1.6–1.7 mm vs. 5.5–6.5 × 3 mm). The flowers are transparent yellow, with a rose to purple suffusion (vs. brown), the lip is triangular-ovate (vs. oblong) the margins being shortly glandular (vs. denticulate), slightly raised but lacking a central sulcus (vs. prominently sulcate).

#### Description.

Epiphytic, caespitose, strictly prolific, erect to suberect herb, up to 30 cm long. ***Roots*** flexuous, thin, ca. 1 mm in diameter, densely spaced, appearing fasciculate. ***Primary ramicauls*** erect to suberect, slender, 7.5–26.0 cm long, with 2 basal tubular, tightly adpressed, papyraceous sheaths, up to 2.0–6.0 cm long. ***Secondary ramicauls*** 1.5–8.0 cm long, produced profusely from the floral meristem on the apex of prior ramicauls, being able to produce clumps and chains of multiple ramicauls before severing naturally. ***Leaves*** spreading to pendent, coriaceous, sessile, narrowly ovate to cordate, acute, blades of primary ramicauls 4.0–7.7 × 1.8–5.0 cm, blades on secondary ramicauls 2.5–5.2 × 1.0–2.5 cm. ***Inflorescence*** persistent, forming successive multi-flowered coflorescences with a single open flower, subtended by a nearly prostrate or suberect spathe which appears deeply torn over time, ca. 1 cm long; pedicels cylindrical. ***Ovary*** slightly clavate, ca. 3 mm long, green, with a few black dots and occasional crystals. ***Flowers*** spreading, transparent yellow, with a rose to purple suffusion of varying intensity, especially observed on the petals, lip, column, and anther cap. ***Dorsal sepal*** narrowly ovate to elliptic, acute, 3-veined, 6.8–7.0 × 3.3–3.4 mm. ***Lateral sepals*** connate in an ovate synsepal, acute, 4-veined, 5.5–6.5 × 4.1–4.2 mm. ***Petals*** naturally drooping, linear-lanceolate, oblique, acute, 1-veined, 5.1–5.4 × 0.6–0.7 mm, glandular, with the margins erose. ***Lip*** triangular-ovate, resting on the synsepal, 2.4–2.6 × 1.6–1.7 mm, blade glandular-papillose, margins minutely glandular, slightly raised, acute, shortly apiculate, with a pair of shoulder-like basal lobes. Glenion a shallow, oblong cavity between raised lateral margins. ***Column*** straight, transversely suboblong, ca. 1.5 mm long, with a short column foot, the anther and stigma apical. ***Anther cap*** ovate, cucullate, obtuse, 2-celled. ***Pollinarium*** composed of 2 narrowly ovate-pyriform pollinia connected to a liquid, drop-like viscidium. ***Fruits*** and ***seeds*** unknown. This description is based on *A.P. Karremans et al. 7600, A.P. Karremans et al. 9135, D. Bogarín et al. 8632, D. Bogarín et al. 12131* and *I. Chinchilla et al. 2865*.

#### Additional specimens examined.

**Figure 6.**
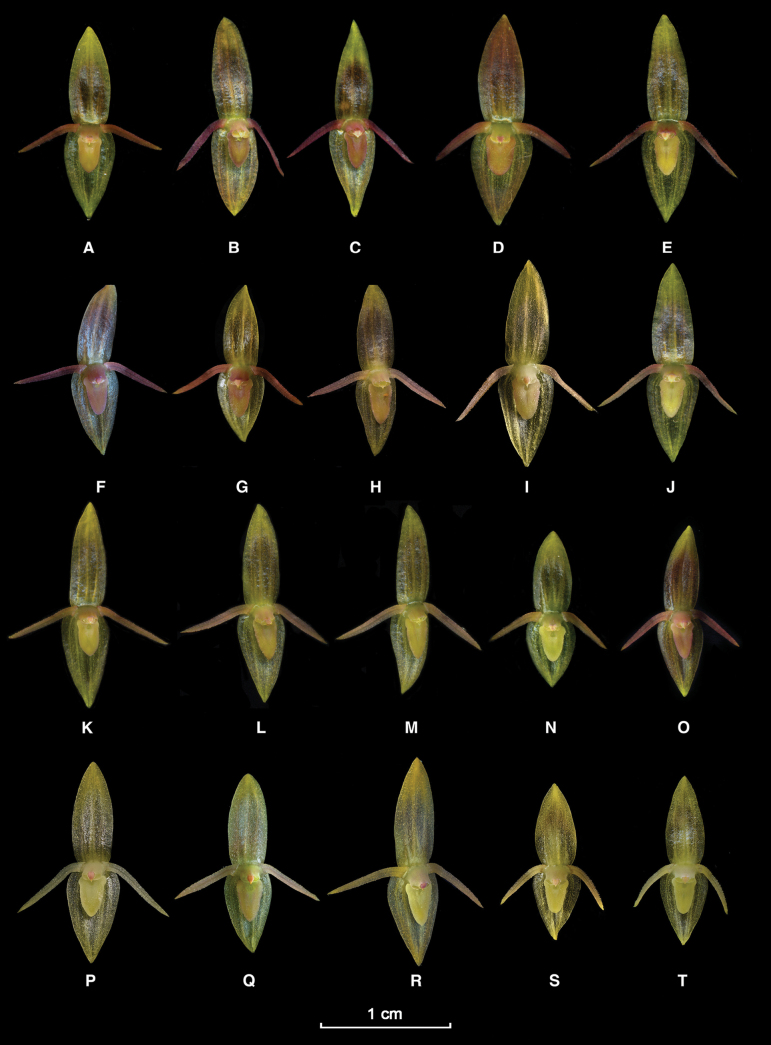
Floral variation in *Pleurothallispridgeoniana***A***Fernández 385***B***Bogarin 7742***C***Karremans s.n.***D***Bogarín 7743***E***Bogarín 7718***F***Bogarín 9806***G***Fernández 382***H***Bogarin 7718***I***Karremans 2829***J***Pupulin 7893***K***Pupulin 7910***L***Fernández 382***M***Karremans 5685***N***Bogarin 7718***O***Bogarin 9806***P***Karremans s.n*. **Q***Pupulin 7887***R***Chinchilla 5865***S, T***Diaz 305*. Photographs by the authors.

Costa Rica-Panama. • Puntarenas-Chiriquí: Coto Brus-Renacimiento, línea fronteriza entre Cerro Quijada del Diablo y Cerro Pando, entre mojones N.336–338, 8°54'51.9"N, 82°43'59.13"W, 2205 m, bosque muy húmedo premontano, epífita en bosque primario, “*in itinere per limitem Costa Rica et Panama inter montis Quijada del Diablo et montis Pando*”, 19 April 2011, fl. in cult. 14 March 2022, *D. Bogarín, D. Jiménez & A.P. Karremans 8632* (JBL-spirit, E1542!, Fig. 5B). Costa Rica-Panama. • Puntarenas-Chiriquí: Coto Brus-Renacimiento, línea fronteriza entre Cerro Quijada del Diablo y Cerro Pando, entre mojones N.336–338, 8°54'51.9"N, 82°43'59.13"W, 2205 m, bosque muy húmedo premontano, epífita en bosque primario, “*in itinere per limitem Costa Rica et Panama inter montis Quijada del Diablo et montis Pando*”, 19 April 2011 fl. in cult. 8 October 2021, *D. Bogarín 8637, D. Jiménez & A.P. Karremans* (JBL-spirit, A0558!). Costa Rica. • Puntarenas: Coto Brus, Sabalito, Zona Protectora Las Tablas, 13 km al noreste de Lucha, Sitio Coto Brus, entre Río Surá y Quebrada Sutú, Finca de Miguel Sandí, 8°56'46.1"N, 82°44'30.9"W, 1778 m, bosque pluvial montano bajo, epífitas en potreros arbolados, 6 June 2010, *A.P. Karremans 2829 & D. Bogarín* (Fig. 6I). • Puntarenas: Buenos Aires, Buenos Aires, camino a Cerros Utyum, 9°18'04.93"N, 83°12'51.59"W, 2157 m, bosque pluvial montano bajo, epífitas en bosque secundario, 16 January 2017, *D. Bogarín, M. Cedeño, I. Chinchilla, M. Díaz, E. Kaes, A.P. Karremans, P. Lehmann & O. Zúñiga 12131* [JBL-spirit, A0375! (fl. in cult. 9 May 2019), A0877! (fl. in cult. 21 March 2023)]. • Puntarenas: Buenos Aires, Buenos Aires. Olán, en el sendero de la Transutyum, después del arbolado, 9°17'56.67"N, 83°12'54.12"W, 2107 m, bosque pluvial montano bajo, bosque primario, epífita, a media luz, 16 January 2017, *I. Chinchilla, D. Bogarín, A.P. Karremans, M. Díaz-Morales, M. Cedeño & E. Kaes 2865* [JBL-spirit, A0187! (fl. in cult. 19 December 2018), K0154! (fl. in cult. 18 agosto 2021), E1543! (fl. in cult. 14 marzo 2022), Fig. 6R]. • Puntarenas: Buenos Aires, Potrero Grande, Altamira, Parque Internacional La Amistad, Sector Altamira, sendero al Valle del Silencio, base del Cerro Hoffmann, 9°05'05"N, 82°58'42"W, 2450 m, 15 May 2022, *A.P. Karremans, I. Chinchilla, L. Oses, G. Rojas-Alvarado & F. Vargas Acuña 9135* [JBL-spirit, A0934! (fl. in cult. 25 July 2024), A0937! (fl. in cult. 25 July 2023), K0412! (fl. in cult. 3 April 2024)]. • Puntarenas: Coto Brus, Sabalito, Zona Protectora Las Tablas, 13 km al noreste de Lucha, Sitio Coto Brus, entre Río Surá y Quebrada Sutú, Finca de Miguel Sandí, 8°56'46.1"N, 82°44'30.9"W, 1778 m, bosque pluvial montano bajo, epífitas en potreros arbolados, 6 June 2010, *D. Bogarín & A.P. Karremans 7718* (Fig. 6E, H, N). Same locality and date, *D. Bogarín 7742 & A.P. Karremans* (Fig. 6B). Same locality and date, *D. Bogarín 7743 & A.P. Karremans* (Fig. 6D). • Límite entre Limón y Puntarenas: Talamanca-Buenos Aires, Bratsi-Potrero Grande, Parque Internacional La Amistad, Sector Altamira, sendero al Valle del Silencio, Cerro Hoffman, sobre la divisoria de aguas, 9°05'38.2"N, 82°58'37.73"W, 2553 m, bosque pluvial montano, 14 August 2012, fl. in cult. 7 February 2019, *D. Bogarín 9806, M. Fernández, J. Godínez, A.P. Karremans, J. Kruizinga & C. Smith* (JBL-spirit, A0216!, Fig. 6F, O). • Puntarenas: Coto Brus, Sabalito, Zona Protectora Las Tablas, 13 km al noreste de Lucha, Sitio Coto Brus, 8°56'46.1"N, 82°44'30.9"W, 1778 m, finca “El Capricho” de Miguel Sandí, principalmente en árboles de *Quercus* en las lomas y potreros al margen del río Sutú, bosque muy húmedo premontano, 6 October 2010, *M. Fernández 382, R.L. Dressler, D. Bogarín & F. Pupulin* [JBL-spirit, D5618!, A0209! (fl. in cult. 5 February 2019), Fig. 6G, L]. Same locality and date, *M. Fernández 385, R.L. Dressler, D. Bogarín & F. Pupulin* (Fig. 6A). • Puntarenas: Buenos Aires, Buenos Aires, Olán, camino a los cerros Utyúm en bosque maduro, 9°18'04.54"N, 83°12'48.72"W, 2129 m, bosque pluvial montano bajo, 16 January 2017, *M. Díaz 305, D. Bogarín, M. Cedeño, I. Chinchilla, A.P. Karremans, P. Lehmann y O. Zúñiga* [JBL-spirit, A0560! (fl. in cult. 8 October 2021), A0575! (fl. in cult. 3 November 2021), Fig. 6S–T]. • Puntarenas: Buenos Aires, Potrero Grande, Altamira, Parque Internacional La Amistad, Sector Altamira,, sendero al Valle del Silencio, ca. 1 km después de Cerro Quemado, 9°04'38.7"N, 82°58'37.4"W, 2284 m, bosque pluvial montano, 14 August 2012, *A.P. Karremans 5685, D. Bogarín, M. Fernández, J. Godínez, J. Kruizinga & C.M. Smith* (Fig. 6M). • Puntarenas: Coto Brus, Sabalito, Zona Protectora Las Tablas, 13 km NE of Lucha, Sitio Coto Brus, finca Sandí “El Capricho”, 8°56'46.1"N, 82°44'30.9"W, 1778 m, epiphytic, mostly on *Quercus* sp. in pastures and along the river Sutú, wet premontane forest, 6 October 2010, *F. Pupulin 7887, D. Bogarín, R.L. Dressler & M. Fernández* (Fig. 6Q). Same locality and date, fl. in cult. 6 December 2012, *F. Pupulin et al. 7910* (JBL-spirit, D5640!, Fig. 6K). Same locality and date, *F. Pupulin et al. 7893* (JBL-spirit, Fig. 6J). • Puntarenas: Coto Brus, Sabalito, Finca Las Tinieblas, propiedad de Billen Gamboa, filas hacia el norte del potrero principal. 8°55'25.6"N, 82°44'55.1"W, altitud 1975 m. Bosques maduros dominados por *Quercus* spp. 10 December 2023, fl. in cult. 9 September 2024, *L. Álvarez 1404, B. Gamboa, M. Mata-Quirós, G. Ramírez, F. Rodríguez, J. H. Flores* (JBL-spirit!).

#### Eponymy.

Honoring the English botanist Alec M. Pridgeon, renowned worldwide for his seminal research on orchid anatomy and phylogenetics, including Pleurothallidinae. A founding editor of the journal *Lindleyana* and main editor of the monumental series of books *Genera Orchidacearum*, which laid the foundation for the contemporary understanding of orchid relationships.

#### Phenology.

Plants in cultivation have been recorded in flower virtually year-round, with flowering peaks in November and February, coinciding in Costa Rica with the end of the rainy season and the beginning of the dry season.

#### Distribution and ecology.

Currently known only from southern Costa Rica and western Panama, where plants grow either epiphytically or terrestrially on organic matter in oak forests at high elevations, around 1800–2550 m. *Pleurothallispridgeoniana* is locally abundant at high elevations on the Cordillera de Talamanca, in the Puntarenas (Costa Rica) and Chiriquí (Panama) provinces respectively. This notoriously prolific species is often found forming large pending mats or clumps in primary and mature forests.

#### Notes.

**Figure 7.**
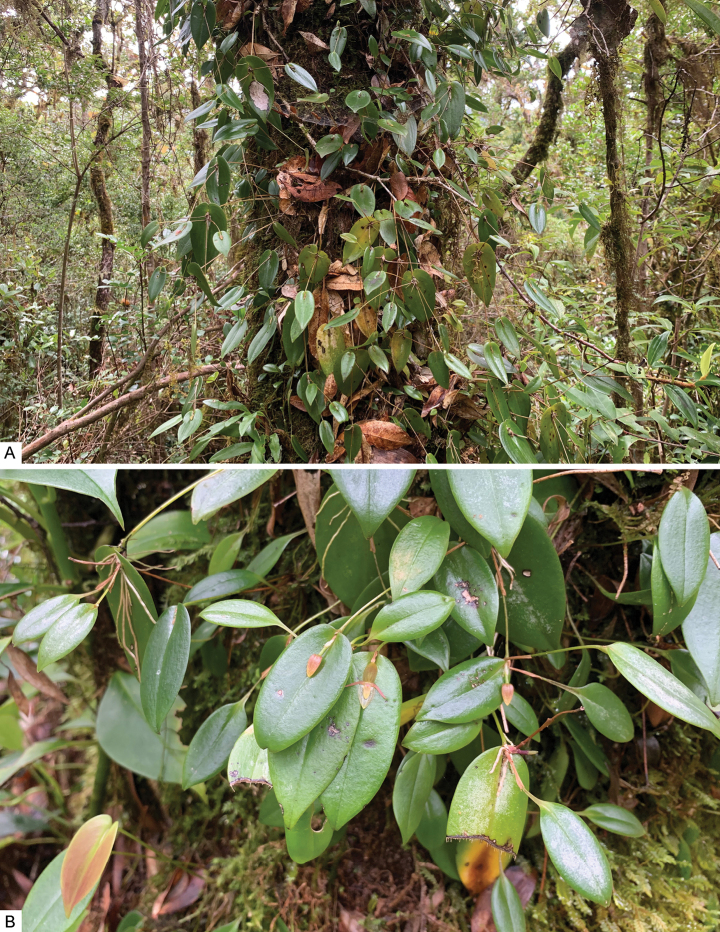
*Pleurothallispridgeoniana* Karremans, Bogarín & Pupulin in its natural habitat in Valle del Silencio on the southern Talamanca range in Costa Rica, close to the border with Panama **A** general overview of the prolific growth habit and asexual production of new units **B** detail of a flowering plant. Photographs by APK.

*Pleurothallispridgeoniana* is easily recognized by the large bushy habit derived from constitutive prolification, in which each ramicaul eventually produces vegetative growths apically, thus originating chains of multiple ramicauls (Fig. 7). The new species seems to be vegetatively most similar to *P.vinealis* from Colombia and Ecuador, which Luer (2005) distinguished by its unique vine-like habit, with prolific ramicauls that twine and branch, apparently attaining more than a meter in length. From *P.vinealis*, *P.pridgeoniana* can be distinguished by the significantly shorter plants, up to 30 cm long (vs. exceeding a meter), the shorter ramicauls 7.5–26.0 cm (vs. up to 40 cm long), the significantly smaller flowers, dorsal sepal 6.8–7.0 × 3.3–3.4 mm (vs. 11–14 × 4.5–5.5 mm), synsepal 5.5–6.5 × 4.1–4.2 mm (vs. 11–14 × 4.5–5.5 mm), lip triangular-ovate (vs. oblong), 2.4–2.6 × 1.6–1.7 mm (vs. 5.5–6.5 × 3 mm). The flowers are transparent yellow, with a rose to purple suffusion (vs. brown), the lip is triangular-ovate (vs. oblong) the margins glandular (vs. denticulate), slightly raised but lacking a central sulcus (vs. notably sulcate).

Florally, the non-prolific *Pleurothallisapplanata* Luer & Dalström and *P.undulata* Poepp. & Endl. (following Luer 2005) are reminiscent of *P.pridgeoniana*. The new species can be distinguished from the former, a species from Ecuador and Peru, by longer ramicauls (up to 26 vs. 12 cm), the smaller flowers, with shorter sepals (5.5–7.0 vs. 9.5–10.0 mm long), narrower (0.6–0.7 vs. 1 mm long), glandular petals, with erose margins (vs. smooth, entire), the shorter and narrower (2.4–2.6 × 1.6–1.7 vs. 4.5 × 2.2 mm) lip, shortly apiculate (vs. obtuse), and erect, subrectangular column, (vs. dorsally compressed on a plane with the lip). From the latter, endemic to Peru, it can be distinguished by the shorter (5.5–6.5 vs. 6–9 mm) synsepal, naturally drooping petals (vs. horizontal to elevated), and the shorter lip (2.4–2.6 vs. 3.0–4.0 mm), with a pair of shoulder-like basal lobes (vs. lip unlobed).

### ﻿ Pleurothallis winkeliana

Taxon classificationPlantaeAsparagalesOrchidaceae

Karremans, Bogarín & Pupulin sp. nov.

07227E51-075C-56C5-99C4-C8EC5704B31E

urn:lsid:ipni.org:names:77362477-1

#### Type.

**Figure 8.**
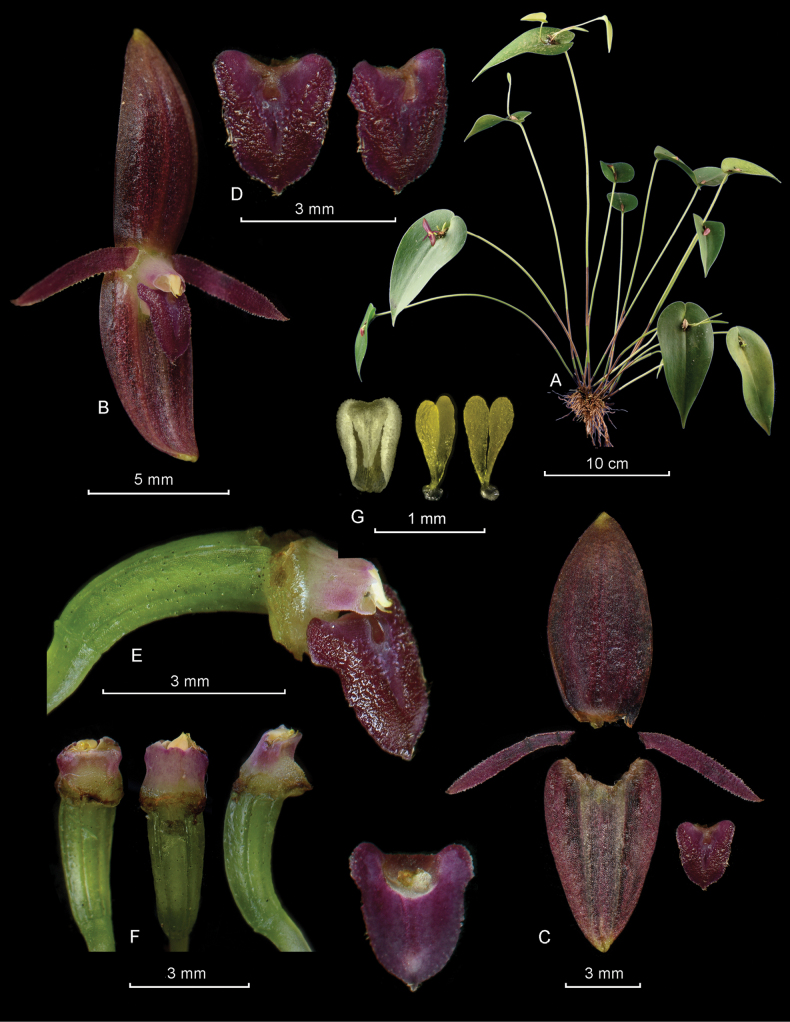
*Pleurothalliswinkeliana* Karremans, Bogarín & Pupulin **A** habit showing some prolific vegetative growths **B** flower **C** dissected perianth (dorsal sepal, two lateral petals, two lateral sepals fused into a synsepal and a view of the lip) **D** two views of the lip **E** column and lip, lateral view **F** column and ovary, ventral, dorsal and lateral views **G** anther cap and two views of the pollinarium. LCDP prepared by FP based on *Karremans 5403*.

**Figure 9.**
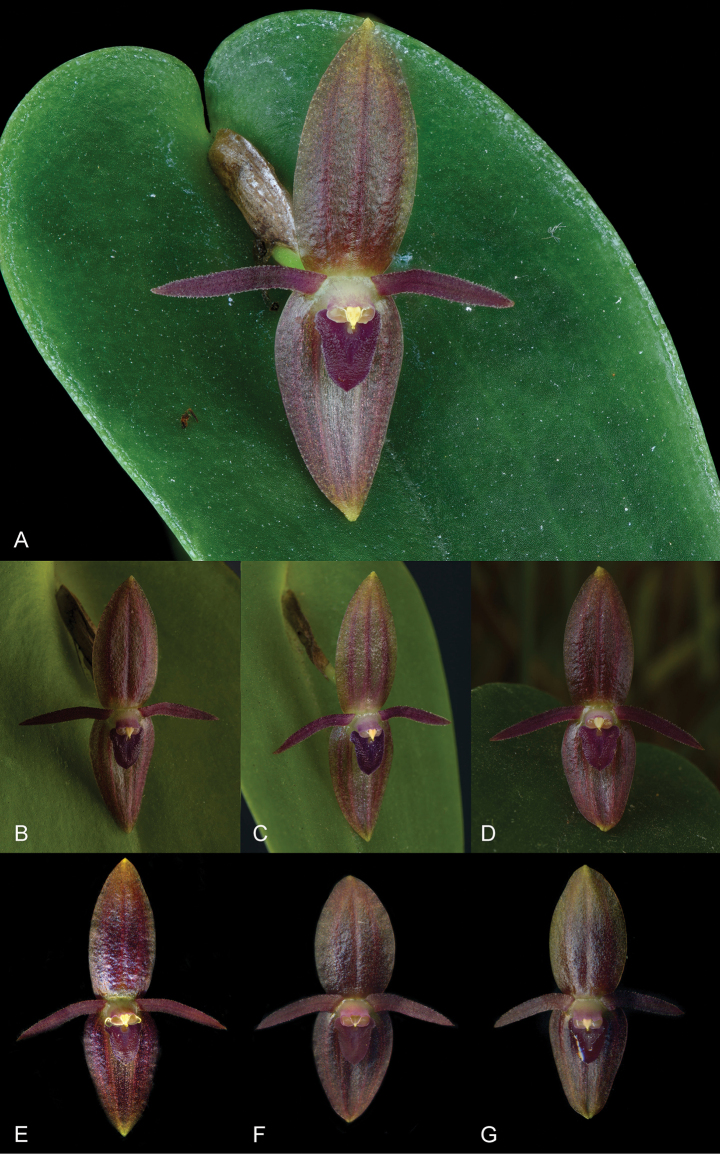
*Pleurothalliswinkeliana* Karremans, Bogarín & Pupulin showing some floral variation **A–E***Karremans 5403***F***Bogarín 9138***G***Bogarín 9140*. Photographs by the authors.

Costa Rica. • Puntarenas: Coto Brus, Sabalito, Zona Protectora Las Tablas, 13 km al noreste de Lucha, Sitio Coto Brus, entre Río Surá y Quebrada Sutú, Finca de Miguel Sandí, 8°56'46.1"N, 82°44'30.9"W, 1778 m, bosque pluvial montano bajo, epífita en potreros arbolados, 20 April 2012, fl. in cult. 25 June 2012, *A.P. Karremans & J. Geml 5403* (holotype: JBL-spirit, E0966!; Figs 8, 9A–E).

#### Diagnosis.

Morphologically closely resembling *P.longipetala* Bogarín & Belfort, but distinguished by the occasionally prolific plant that produces clumps of ramicauls (vs. non-prolific), the proportionally broader (ratio length:width = 2:1 vs 3:1) leaves, cordate in shape (vs. narrowly ovate-lanceolate) with overlapping basal lobes (vs. basal lobes non-overlapping), the smaller flower (<1.5 vs > 2 .0 cm), the shorter and narrower petals (5.4–5.7 × 0.7–0.8 vs. 7.9–8.6 × 1.6–1.7 mm) and by the lip, which is pendent and perpendicular to the column (vs erect, parallel to the column), and obscurely glandular (vs. thickly verrucose).

#### Description.

Epiphytic, caespitose, occasionally prolific, erect to suberect herb, up to 25 cm tall. ***Roots*** flexuous, thin, ca. 1 mm in diameter, densely spaced, appearing fasciculate. ***Primary ramicauls*** erect to suberect, slender, up to 9.0–23.0 cm long, with 2 tubular, tightly adpressed, papyraceous sheaths, up to 2.5–4.3 cm long, one close to the base, and another reaching the middle of the ramicaul. ***Secondary ramicauls*** 1.0–3.5 cm long, produced profusely from the floral meristem on the apex of primary ramicaul, being able to produce clumps of multiple ramicauls before severing naturally. ***Leaves*** horizontal to suberect, coriaceous, sessile, cordate, acute, with the basal lobes confluent to overlapping. Blades of primary ramicauls 4.0–9.6 × 1.5–4.0 cm, blades on secondary ramicauls 1.5–5.4 × 0.6–1.7 cm. ***Inflorescences*** persistent, forming successive multi-flowered coflorescences with a single open flower, subtended by a nearly prostrate or suberect spathe which appears deeply torn over time, ca. 1 cm long; pedicels terete, pale gray-yellow, with irregular black flecks. ***Ovary*** slightly clavate, bent, 3.0–3.2 mm long, green, with occasional black dots. ***Flowers*** spreading, transparent purple, with dark purple petals and lip, a whitish column, suffused with pink, and a yellowish anther cap. ***Dorsal sepal*** elliptic, acute, 3-veined, 8.5 × 4.0–4.2 mm. ***Lateral sepals*** connate in a narrowly ovate synsepal, acute, 4-veined, 7.3–7.8 × 4.7–4.8 mm. ***Petals*** linear, oblique, acute, 1-veined, 5.4–5.7 × 0.7–0.8 mm, with glandular margins. ***Lip*** triangular-ovate, resting on the synsepal, 2.6–2.8 × 2.3 mm, blade glandular, margins glandular-erose, acute, shortly apiculate, glenion a deep, oblong basal cavity. ***Column*** straight, transversely subrectangular, ca. 1.5 mm long, with a very short, glandular column foot, the anther and stigma apical. ***Anther cap*** ovate, cucullate, obtuse, 2-celled. ***Pollinarium*** composed of 2, narrowly ovate-pyriform pollinia, connected to a liquid, drop-like viscidium. ***Fruits*** and ***seeds*** unknown. This description is based on *A.P. Karremans & J. Geml 5403*.

#### Additional specimens examined.

Costa Rica. • Limón: Talamanca, Telire. Cordillera de Talamanca, Parque Internacional La Amistad (ACLA-C), sendero de la transtalamanca, bajando de la Fila Bugú hacia el Río Tapari, 09°26'48.00"N, 83°11'12.00"W, 1380 m, bosque pluvial premontano, bosque primario, epífita, a media luz, collected 27 April 2017, *I. Chinchilla 3408, A.P. Karremans, G. Rojas-Alvarado, M. Cedeño, E. Kaes & O. Zúñiga* [JBL-spirit, J0784! (prepared 14 May 2018), A0366! (prepared 2 May 2019)]. • Puntarenas: Buenos Aires, Potrero Grande, Altamira, Parque Internacional La Amistad, Sector Altamira, sendero al Valle del Silencio, cerca Cerro Hoffman, 9°04'56.2"N, 82°58'36.4"W, 2347 m, bosque pluvial montano, 30 August 2011, *D. Bogarín 9138 & A.P. Karremans* (Fig. 9F). Same locality and date, *D. Bogarín 9140 & A.P. Karremans* (Fig. 9F). • Puntarenas: Coto Brus, Sabalito, Zona Protectora Las Tablas, 13 km al noreste de Lucha, Sitio Coto Brus, entre Río Surá y Quebrada Sutú, Finca de Miguel Sandí, 8°56'46.1"N, 82°44'30.9"W, 1778 m, bosque pluvial montano bajo, epífita en potreros arbolados, collected 20 April 2012, *A.P. Karremans & J. Geml 5403* (JBL-D6595! (prepared 9 January 2015), JBL-A0237! (prepared 29 November 2018), JBL-A0250! (prepared 21 February 2019), JBL-J1024! (prepared 12 July 2016).

#### Eponymy.

Honoring Dutch botanist Gab van Winkel (1955–2023), late editor of Orchideeën, journal of the Dutch Orchid Society (Nederlandse Orchideeën Vereniging), and director of the official website of the European Orchid Council (EOC). Gab has been recognized for devoting his life to the study of orchids (Anghelescu et al. 2024), and his untimely passing has been a great loss.

#### Phenology.

Flowering of *P.winkeliana* has been recorded from November to July, mostly corresponding to the dry season in Costa Rica.

#### Distribution and ecology.

Currently known only from the southern Cordillera de Talamanca in Costa Rica, where plants grow epiphytically in primary forests at around 1400–2300 m of elevation. *Pleurothalliswinkeliana* appears to be rare but is found on both watersheds of the Cordillera de Talamanca in southern Costa Rica, close to the Panamanian border, where it has been recorded in the neighboring Limón and Puntarenas provinces.

#### Notes.

*Pleurothalliswinkeliana* belongs to the *P.phyllocardia* assemblage (sensu Pupulin et al. 2021), characterized by presenting mostly narrow leaves, an erect to suberect, non prostrate spathaceous bract [except in *P.adventurae* Karremans & Bogarín (Karremans and Bogarín 2011: 112)], and coriaceous flowers that remain open after anthesis. In Costa Rica, this assemblage includes 13 species and a natural hybrid. *Pleurothalliswinkeliana* is most similar to *P.longipetala*, which was described from Tapantí in central Costa Rica, growing at an elevation of 1453 m. From the latter, *P.winkeliana* is mostly distinguished by the taller habit, and by its frequently prolific stems, that are topped by comparatively broad (ratio length:width = 2:1 vs 3:1) cordate leaves (vs. narrowly ovate-lanceolate), which bear overlapping (vs. non-overlapping) basal lobes. The flowers of *Pleurothalliswinkeliana* are smaller, with the smaller petals (5.4–5.7 × 0.7–0.8 vs. 7.9–8.6 × 1.6–1.7 mm), distinctly narrower at the base (vs. broadened at the base), and the lip is geniculate at the base and held perpendicularly to the column (vs. straight, parallel), with the blade obscurely glandular (vs. thick verrucose). The new species is also reminiscent of *Pleurothallisanthurioides* A.Doucette from Costa Rica and Panama (Pupulin et al. 2021), but the flowers are much smaller (e.g. sepal length 7.3–8.5 vs. 12–19 mm, petals 5.4–5.7 × 0.7–0.8 vs. 10–13 × 3–4 mm), the petals are linear (vs. oblong) and the lip triangular-ovate (vs. oblong-peltate).

## ﻿Discussion

Despite occurring across the Pleurothallidinae (Figs 1, 2), prolification is a poorly studied phenomenon. The three new taxa described here differ slightly in their means of becoming prolific. *Pleurothallismatrisilvae* is typically prolific when growing as an epiphyte, while being mostly non-prolific when found growing on top of organic matter on the ground. *Pleurothallispridgeoniana* shows constitutive prolific growth, forming large, bushy plants, with each ramicaul eventually producing vegetative growths apically, resulting in chains of multiple ramicauls. *Pleurothalliswinkeliana* often forms clumps, rather than chains, of ramicauls at the apex of some, but not all, primary ramicauls. Be it constitutive or facultative, in the case of the genus *Pleurothallis*, prolification occurs more frequently in individuals and species growing at higher elevations.

Recent studies indicate that the highest rates of orchid speciation in the Neotropics occur in the humid and seasonal forests of Costa Rica and Panama (Pérez-Escobar et al. 2024). Specifically, Pleurothallidinae, which is the most species-rich group in this region, has experienced a rapid and recent radiation, reflected in a high diversity in Costa Rica (Pérez-Escobar et al. 2017). However, biogeographic studies on Pleurothallidinae are still limited due to incomplete species sampling and the reliance on a few molecular markers that exhibit low variability in previous studies (Pérez-Escobar et al. 2017). The sampling of multiple nuclear and plastid markers remains low within the subtribe, and it has not yet been tested whether including hundreds of markers could help resolve recalcitrant nodes in the phylogenetic analyses, specifically within *Pleurothallis* (Bogarín et al. 2018; Chumová et al. 2021), and thus clarify the exact phylogenetic placement of taxa in species-rich and non-monophyletic groups such as Pleurothallissubsect.Macrophyllae-*Fasciculatae*, which includes the three novelties.

**Figure 10.**
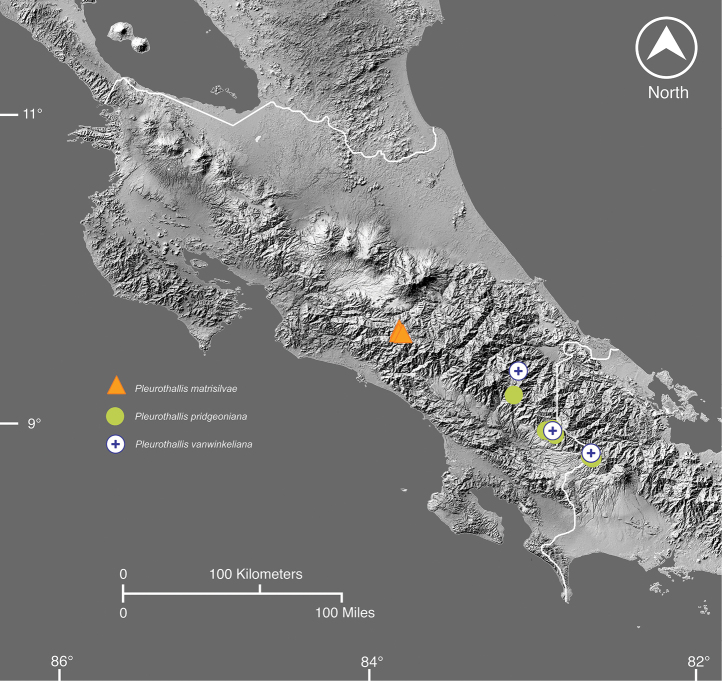
Distribution map of *P.matrisilvae*, *P.pridgeoniana*, and *P.winkeliana* based on field collections.

The description of the three new *Pleurothallis* brings the total for the genus in Costa Rica to 67 species and four natural hybrids. In Costa Rica, *Pleurothallis* includes several species complexes that represent local radiations for which multiple species still require recognition, despite recent efforts. Novelties are often found on the highly diverse and largely unexplored Talamanca range in central and southern Costa Rica (Fig. 10).

To date, detailed analyses of species distributions have not been conducted, partly due to unresolved taxonomic issues and the lack of accuracy in species identification within many diverse or understudied genera of Pleurothallidinae, such as *Lepanthes*, *Pleurothallis*, and *Stelis*. Nevertheless, data available on *Pleurothallis* suggest distinct distribution patterns among Costa Rica’s mountain ranges. For instance, *P.chavezii* Luer is found only in the Cordillera de Guanacaste, while *P.adventurae* Karremans & Bogarín, *P.anthurioides*, and *P.maduroi* Luer are exclusive to the Cordillera de Talamanca. In contrast, other species, such as *Pleurothallistonduzii* Schltr., have a broader distribution across all mountain ranges. Meanwhile, *P.angusta* Ames & C.Schweinf., and *P.phyllocardia* Rchb.f. are widespread in the Cordillera de Talamanca and Cordillera Central but do not extend to the Cordillera de Tilarán or Guanacaste (Pupulin et al. 2021). No collections of the three novelties described herein have been found in other mountain ranges of Costa Rica, suggesting that their distribution may be restricted to the Cordillera de Talamanca. Based on previous observations of other Pleurothallidinae species, we do not expect to find these newly described species in the northern mountain ranges of Guanacaste and Tilarán.

A comprehensive study comparing the diversity of orchids across Costa Rica’s mountain ranges has not yet been conducted. By completing the ongoing taxonomic work on these complex and diverse groups within *Pleurothallis*, integrating new distribution data from recently described species, we can establish a stronger foundation for more accurate biogeographical analyses.

## Supplementary Material

XML Treatment for Pleurothallis matrisilvae

XML Treatment for Pleurothallis pridgeoniana

XML Treatment for Pleurothallis winkeliana

## ﻿Additional information

### Author ORCIDs

Adam P. Karremans https://orcid.org/0000-0001-5987-7710

Franco Pupulin https://orcid.org/0000-0001-6780-8395

John Gange https://orcid.org/0009-0008-2868-9248

Diego Bogarín https://orcid.org/0000-0002-8408-8841

### Data availability

All of the data that support the findings of this study are available in the main text.
